# Largely Reduced Grid Densities in a Vibrational Self-Consistent Field Treatment Do Not Significantly Impact the Resulting Wavenumbers

**DOI:** 10.3390/molecules191221253

**Published:** 2014-12-17

**Authors:** Oliver M. D. Lutz, Bernd M. Rode, Günther K. Bonn, Christian W. Huck

**Affiliations:** 1 Institute of Analytical Chemistry and Radiochemistry, Center for Chemistry and Biomedicine, University of Innsbruck, Innrain 80-82, Innsbruck 6020, Austria; E-Mails: guenther.bonn@uibk.ac.at (G.K.B.); christian.w.huck@uibk.ac.at (C.W.H.); 2 Institute of General, Inorganic and Theoretical Chemistry, Center for Chemistry and Biomedicine, University of Innsbruck, Innrain 80-82, Innsbruck 6020, Austria; E-Mail: bernd.m.rode@uibk.ac.at

**Keywords:** VSCF, grid density, spectroscopy, anharmonicity, MP2, infrared, vibrational self-consistent field, phosphoserine

## Abstract

Especially for larger molecules relevant to life sciences, vibrational self-consistent field (VSCF) calculations can become unmanageably demanding even when only first and second order potential coupling terms are considered. This paper investigates to what extent the grid density of the VSCF’s underlying potential energy surface can be reduced without sacrificing accuracy of the resulting wavenumbers. Including single-mode and pair contributions, a reduction to eight points per mode did not introduce a significant deviation but improved the computational efficiency by a factor of four. A mean unsigned deviation of 1.3% from the experiment could be maintained for the fifteen molecules under investigation and the approach was found to be applicable to rigid, semi-rigid and soft vibrational problems likewise. Deprotonated phosphoserine, stabilized by two intramolecular hydrogen bonds, was investigated as an exemplary application.

## 1. Introduction

The continuously increasing availability of computational resources as well as the development of accurate and efficient quantum chemical approaches have made computational vibrational spectroscopy an indispensable field complementing experimental techniques. Nowadays, almost every quantum chemical software package enables the analysis of second-order properties of the energy (e.g., IR and Raman absorptions, IR intensities, electric dipole polarizabilities, nuclear magnetic resonance chemical shifts, spin-spin coupling constants). Arguably, the prediction of IR and Raman absorptions is among the most important applications considering the prevalence of these technique. While most fundamental IR absorptions can be assigned quite satisfyingly on an empirical basis or via normal coordinate analysis, especially the near-infrared region is cluttered by overtones and combination excitations that are cumbersome to assign.

With regard to computational approaches, the harmonic oscillator approximation (HOA) is the most fundamental technique for obtaining vibrational spectroscopic data. However, due to the rigorous assumption introduced, in that the bond potential exhibits a harmonic shape, significant deviation from experiment is frequently observed. The most simple solution accounting for the lack of anharmonicities is to introduce empirical scaling factors that are multiplied with the harmonically approximated absorptions. Scott and Radom derived scaling factors for a vast number of *ab initio* and semi-empirical methods and a large number of basis sets [1,2]. It has to be stressed, however, that an empirical scaling may not be applied on a system-independent basis, even though the scaling factors have been derived for a rather large set of molecules. Noteworthy, a harmonic bond potential cannot be assumed a proper basis for considerations towards excitations involving more than one quantum of energy.

Especially due to these fundamental deficiencies, further corrective techniques have been developed that account for anharmonicities in an explicit manner. The two most prominent approaches are the vibrational self-consistent field (VSCF) method [3,4,5,6,7,8] and the vibrational second order perturbative ansatz (VPT2) [9,10]. VPT2 is a technique that relies on the computation of higher-order derivatives of the energy. Gaussian [11] is probably the most prominent commercial software package incorporating a VPT2 algorithm which relies on third and semi-diagonal fourth derivatives of the energy with respect to the nuclear coordinates. This method is applied regularly [12,13] but it may only be employed safely to vibrational problems where the harmonic part of the potential is dominant. Moreover, the computationally demanding generation of third and fourth derivatives limits the approach to rather small systems. VSCF on the other hand utilizes a separability ansatz which enables the anharmonicity of each mode to be accounted for by screening the respective potential in a point-wise manner. Mode interactions may be evaluated by computing *d* dimensional grids and the maximum possible dimensionality is determined by the number of vibrational degrees of freedom (*N* ) featured by the molecule under investigation. In a conventional VSCF calculation, each mode’s potential is characterized by 16 grid points [14]. In the past, scientific papers have been reported that employed grids of higher (*r* = 32) [15] and lower resolutions (*r* = 8) [16,17,18]. Roy *et al.* [19] recently presented data based on a variety of grid densities ranging from 8 to 16 points. Since they reported overall deviations summed over a set of reference molecules, one could conclude that this rather diverse choice of grid resolutions does not impose a significant error on the wavenumbers. However, to date there still seem to exist ambiguities with regard to a generally applicable grid density that is computationally feasible. Hence, and considering the massive computational effort involved in high resolution VSCF calculations, it seemed promising to conduct a systematic study addressing these issues. 15 reference molecules are investigated in detail and an application to the lowest energy conformer of phosphoserine is presented. At this point it seems worth mentioning that while many quantum chemical software packages such as GAMESS [20], NWChem [21] and MOLPRO [22] incorporate VSCF implementations, GAMESS is the program employed in this work due to the fact that it is freely available and compatible with the most prominent computer operating systems.

## 2. Methods

In this section, a brief overview describing the procedures underlying a VSCF evaluation is given. Particularities of the involved techniques are presented while reference to the original literature is given for further details.

### 2.1. Energy Minimization

A prerequisite for every computational spectroscopic analysis is a proper structural ensemble. For the majority of cases, the user would want to obtain absorption data corresponding to an equilibrium structure and thus, an energetically favorable reference geometry is required. This implies that at least a local energy minimum (or the global energy minimum) is obtained and this is reflected by a nearly zero gradient of the energy *g*(*q*)∆*q* with respect to the nuclear coordinates *q*. While the program’s default criteria suffice for energetical and structural considerations in many cases, a VSCF calculation requires a thoroughly minimized geometry. This condition is realized by setting up rigorous cutoff tolerances for *g*(*q*)∆*q*. We considered a molecular geometry properly minimized when each gradient contribution is smaller than 0.000001 E_*h*_· Bohr^−1^. Every calculation reported herein was performed at the Møller-Plesset level of theory, accounting for electron correlation effects via a perturbative ansatz [23,24,25]. The frozen-core approximation [26] was employed, explicitly correlating all but the core electrons. Dunning’s correlation-consistent polarized basis set of triple-*ζ* quality has been utilized since it can be considered a routinely employed set of functions that is known to deliver results of acceptable accuracy [27]. The appropriate point group was imposed onto the molecular geometry where applicable in order to make use of the efficient symmetry optimized SCF algorithm in GAMESS.

### 2.2. Second Derivatives of the Energy

A potential *V*(*q*) may be expressed as a Taylor series

(1) $$V(q)=V_{0}(q)+g(q)\Delta q+\frac{1}{2}\Delta q^{T}ℍ\Delta q+\ldots$$

and for the case that the reference geometry resembles an energy minimum and assuming a harmonic potential shape, all but the first and third terms vanish. This simplified approach enables the evaluation of the elements of ℍ_*r,s*_ by analytical or numerical analysis:

(2) $${ℍ}_{r,s}=\frac{\partial^{2}V(q)}{\partial q_{r}\partial q_{s}}$$

A transformation into mass-weighted coordinates and diagonalization of the Hessian matrix ℍ_*r,s*_ yields the eigenvectors corresponding to the normal modes. At this point, computational spectroscopic data at the HOA level is available. It seems worth mentioning that the further description of extended vibrational analysis is based on mass-weighted normal coordinates or other non-redundant coordinates for the sake of clarity, whereas the expansion of the potential energy surfaces was realized in rectilinear coordinates (Section 3.1). VSCF calculations require a Hessian corresponding to the equilibrium geometry, as will be discussed in the next paragraph. The Hessian matrices in this work have been obtained semi-numerically involving two displacements of ±0.01 Bohr about the reference geometry for each atom.

### 2.3. The VSCF Routine

Even though VSCF accounts for anharmonicities explicitly, it still requires ℍ_*r,s*_ as a reference state. This is owed to the fact, that VSCF assumes a molecule’s vibrational wave function Ψ(*Q*_1*,..,N*_) to be separable into single mode wave functions $\psi_{i}^{\left(n\right)}$:

(3) $$\Psi (Q_{1,\ldots ,N})=\prod_{i=1}^{N}\psi_{i}^{\left(n\right)}(Q_{i})$$

While this product ansatz can be implemented rather efficiently, it inherently limits the accuracy of the VSCF technique since it represents decoupled vibrational modes. There are, besides the methods mentioned in Section 2.4, techniques available which are able to circumvent this limitation [28,29]. Introducing the variational principle [30,31], the single mode VSCF equation [6,32] is formulated as:

(4) $$\left[-\frac{1}{2}\frac{\partial^{2}}{\partial Q_{i}^{2}}+\bar{V_{i}^{\left(n\right)}}(Q_{i})\right]\psi_{i}^{\left(n\right)}(Q_{i})=\varepsilon_{i}^{\left(n\right)}\psi_{i}^{\left(n\right)}(Q_{i})$$

where $\bar{V_{i}^{\left(n\right)}}(Q_{i})$ is mode *Q*_*i*_’s effective potential. Equation (4) neglects parts of the full Watson Hamiltonian for non-linear molecules [33] which accounts for *N* vibrational degrees of freedom:

(5) $$Ĥ=-\frac{1}{2}\sum_{i=1}^{N}\frac{\partial^{2}}{\partial Q_{i}^{2}}+V(Q_{1,\ldots ,N})+\frac{1}{2}\sum_{\alpha \beta }{\hat{\pi }}_{\alpha }\mu_{\alpha \beta }{\hat{\pi }}_{\beta }-\frac{1}{8}\sum_{\alpha }\mu_{\alpha \alpha }$$

The second and third part of the Hamiltonian represent the generalized inverse of the effective moment of inertia *µ*_*αβ*_ and the vibrational angular momentum operator ${\hat{\pi }}_{\alpha }$. Given that the full Watson Hamiltonian is cumbersome to implement computationally, most implementations resort to neglecting the last two terms of *Ĥ*. The error introduced is small especially for fundamental excitations as has been shown by Bowman *et al.* [34].

The VSCF equations are solved in an iterative manner until self-consistency is achieved. Illustratively, VSCF may be seen as an approach where each vibration is influenced by the mean field of the surrounding modes. However, especially when certain mode interactions exhibit unique properties, this mean field ansatz does no longer properly describe the vibrational problem sufficiently well. A number of post-VSCF techniques accounting for this deficiency have been presented and they are discussed in the subsequent section.

Since the VSCF technique involves a grid-based screening of bond potentials and their interactions, a discussion of the so-called hierarchical expansion is justified. For a system exhibiting *N* normal modes, the VSCF expansion is denoted as:

(6) $$V(Q_{1,\ldots ,N})=\sum_{i=1}^{N}V_{i}^{diag}(Q_{i})+\sum_{i<j}^{N}V_{i,j}^{pairs}(Q_{i},Q_{j})+\sum_{i<j<k}^{N}V_{i,j,k}^{triples}(Q_{i},Q_{j},Q_{k})+\ldots$$

A complete interaction scheme is realized by imposing *N* -dimensionality onto Equation (6) but obviously, such a treatment scales unfavorably with the studied system size. Noteworthy, most applications of VSCF theory truncate the expansion largely by including only $\sum_{i=1}^{N}V_{i}^{diag}(Q_{i})$ and $\sum_{i<j}^{N}V_{i,j}^{pairs}(Q_{i},Q_{j})$ contributions [35,36,37,38]. This simplification is valid for a large number of applications and the inclusion of higher-order interaction terms is hardly justifiable for other than the smallest molecules or for cases, where convergence is only achieved when including such terms. Programs such as MULTIMODE [34,39], Molpro [22] and MIDAScpp enable coupling orders of *d* > 3 yielding IR bands of exceptional accuracy [7,40]. In this article, we confine our discussion to $\sum_{i=1}^{N}V_{i}^{diag}(Q_{i})$ and $\sum_{i<j}^{N}V_{i,j}^{pairs}(Q_{i},Q_{j})$ contributions since this study aims at larger molecules where an incorporation of higher coupling terms would be prohibitive. The number of grid points due (*N*_*p*_) is calculated via Equation (7) .

(7) $$N_{p}=r\times N+\frac{N\times (N-1)}{2}\times r^{2}$$

Assuming *r* = 16 and taking glycine as an example, the pair-wise approximation would require 71,040 points to be computed while a VSCF calculation involving also three-mode interactions already gives rise to 8,361,344 energy evaluations, a nearly 120 fold increase in computational burden. Herein, we will examine the impact of the grid density on the quality of the absorption data by conducting VSCF calculations involving between 6 and 16 grid points per mode. For the displacements underlying the PES scan, a symmetric grid range of [−4$\omega_{i}^{-0.5}$, +4$\omega_{i}^{-0.5}$], with *ω*_*i*_ being the harmonic frequency of the *i*-th normal mode, was chosen. The grid points within these boundaries have been set-up in an equidistant manner which implies that for even grid densities (*i.e.*, 6, 8, 10, 12, 14 and 16 points), equilibrium is located between the two innermost grid points. For odd grid densities (*i.e.*, 7, 9, 11, 13 and 15 points), equilibrium is described by one distinct grid point. For every resolution *r* < 16, the grid points are interpolated to the original resolution of *r* = 16 by a polynomial fit.

### 2.4. Extensions of the VSCF Approach

As mentioned earlier, the VSCF assumption does not hold for many applications. Therefore, a perturbative approach has been suggested, accounting for the error introduced by the mean-field VSCF ansatz [15,41,42]. A prerequisite is that the difference between the "true" energy and the mean-field VSCF energy is small. An expansion known from electronic structure theory [23] is then formulated for the energy and wave function of a state *n* that is truncated at second order:

(8) $$E_{n}\approx E_{n}^{0}+\lambda E_{n}^{1}+\lambda^{2}E_{n}^{2}$$

(9) $$\Psi_{n}\approx \Psi_{n}^{0}+\lambda \Psi_{n}^{1}+\lambda^{2}\Psi_{n}^{2}$$

with *H* = $H_{n}^{0}$ + *λ∆V* and an insertion of *E*_*n*_ and Ψ_*n*_ in *H* Ψ_*n*_ = *E*_*n*_Ψ_*n*_, the energy contribution at second order may be formulated as:

(10) $$E_{n}^{2}=\sum_{m\neq n}\frac{\langle \Psi_{n}^{0}\left|\Delta V\right|\Psi_{m}^{0}\rangle \langle \Psi_{m}^{0}\left|\Delta V\right|\Psi_{n}^{0}\rangle }{E_{n}^{0}-E_{m}^{0}}$$

Herein, $\Psi_{n}^{0}$ and $\Psi_{m}^{0}$ are the unperturbed vibrational product wave functions and $E_{n}^{0}$ and $E_{m}^{0}$ are the unperturbed VSCF energies. This technique, which is commonly abbreviated as second order perturbation theory augmented (PT2)-VSCF or correlation-corrected (CC)-VSCF, has proven useful for many applications. However, since obtaining correlation-corrected VSCF energies requires significant computational resources, the basic PT2-VSCF approach is limited to small systems. Due to the inherent need for an efficient solution of Equation (10), Gerber’s work group developed a solution based on the assumption of orthogonal vibrational single mode wave functions (Equation (3)) which leads to the annihilation of diagonal elements in Equation (10) [43]. Tests involving *Gly*_*n*_ peptides showed that the runtime improvement is greater for larger molecules: for monomeric *Gly*, a speedup factor close to 6 was observed while the correlation-corrected wavenumbers of tetraglycine have been evaluated more than 16 times faster as with the conventional PT2-VSCF ansatz. This acceleration technique, and also the fact that this accelerated PT2-VSCF technique is readily implemented in GAMESS [20], are probably the main reasons for PT2-VSCF being a routinely employed VSCF correction. It has to be stressed, however, that the evaluation of the VSCF equations with its further corrections may not be confused with the preceding and highly demanding evaluation of the potential energy grid (*vide supra*).

Problems may arise when degenerate vibrational states are present. PT2-VSCF can fail here due to a close to zero denominator in Equation (4) which can lead to a largely overestimated perturbative correction. Hence, the degenerate PT2-VSCF method has been developed [44] but to date it has been implemented in GAMESS exclusively for degeneracies arising from fundamental excitations. A more generally available simple solution available in the GAMESS program code is that contributions involving denominators falling below a critical value are excluded from the treatment. Most applications reporting PT2-VSCF derived data rely on this simplification and therefore, we will also confine our calculations to this simplified technique.

Besides perturbation theory augmented VSCF, significant effort is put into post-VSCF techniques involving configuration interaction [45,46,47,48], coupled-cluster [49,50,51] and multi-configurational SCF theory [52,53]. While such methods yield highly accurate data, they are to date only applicable to small vibrational problems with less than 20 atoms.

Due to the popularity of the PT2-VSCF method and the fact that results of good quality at manageable computational cost are available also for larger molecules, all data presented in this paper is corrected exclusively with this perturbative ansatz.

## 3. Results and Discussion

The fifteen molecules under investigation gave rise to 176 distinct vibrational degrees of freedom, considering all fundamental stretching, deformation and torsional vibrations. PT2-VSCF can yield questionable or sometimes even divergent results for very low-lying and floppy torsions, which is due to the fact that the PES expansion is conventionally carried out in Cartesian coordinates [18,19,54]. The error induced through an anharmonic correction of such a vibration can exceed the boundaries of accuracy known from the HOA [54]. Hence, the usual procedure is to either treat such vibrations harmonically or to describe the underlying displacements in internal coordinates. A substitution for physically more meaningful internal coordinates was proposed by Njegic and Gordon [54] and could be shown to yield good results for formamide and thioformamide [55] as well as for H_2_O_2_ but by introducing a new expansion technique for the kinetic energy operator [56]. Nonetheless, setting up internal coordinates that properly describe the displacements underlying a VSCF treatment is by no means a trivial task. The GAMESS code is able to identify each normal mode’s contributions to particular internal coordinates [57], but the user still has to input a balanced description of each vibrational degree of freedom which can become unmanageably difficult for larger systems. Hence, and especially when large molecules are investigated, the majority of users of VSCF theory resort to a PES expansion in Cartesian coordinates and the contributions of critical torsions are omitted when self-consistency is not achieved.

For glycine (C_2_H_5_NO_2_), three normal modes (*i.e.*, the N-C_*α*_-C_*carb*_-O torsion, the NH_2_ group torsion and *δ*_*C*_*carb*_*O*_2*,oop*__) had to be excluded from the VSCF treatment since they are known to lead to divergence during a perturbation theory corrected VSCF evaluation [19]. Similarly, for methanol (CH_3_OH) the CO axis torsion has been omitted from the PT2-VSCF treatment due to an inadequate description of this particularly floppy torsional mode within the VSCF framework [58]. Dimethylether also exhibits two floppy torsions involving the C-O axes that have been excluded likewise. For ethane, one normal mode near 290 cm^−1^ [59,60,61,62] was omitted due to its floppy character but the other six missing modes did arise from degenerate states in *ν*_*CH*_3_*,as*_, *δ*_*CH*_3_*,as*_ and *ρ*_*CH*_3__. Experimentally, these degeneracies cannot be distinguished and since the PT2-VSCF derived values did not exhibit significant numerical discrepancies, each pair of degeneracies is presented as a single mean value for the sake of visibility.

### 3.1. Performance of the PT2-VSCF Approach

The computationally obtained absorption data are compared to experimental data in Table 1. The column headers indicate the employed number of grid points during the VSCF evaluations. As a measure of quality, the mean absolute percentage error *µ* for each molecule and each grid density is calculated according to Equation (11):

(11) $$\mu =\frac{100}{N}\sum_{i=1}^{N}\left|\frac{{ṽ}_{Expt}^{i}-{ṽ}_{VSCF}^{i}}{{ṽ}_{Expt}^{i}}\right|$$

The obtained values for *µ* are generally smaller than 2%, which is in good agreement with the recent work by Roy *et al.* [19] who concluded that MP2/cc-pVTZ based PT2-VSCF evaluations exhibit a mean unsigned error of under 2%. MP2 is a topical *ab initio* method that accounts for electron correlation effects to a certain extent and it seems as if for most molecules, this method indeed delivers satisfactory results. Acetonitrile is an exception due to its CN triple bond. However, the particularities of this molecule are discussed elsewhere [63] and it was found that state-of-the-art quantum chemical methods [64,65,66,67,68,69] are required for a proper description of *ν*_*CN*_. Importantly, the more or less ubiquitous stretching motions arising from a methyl group are not described in a reliable manner and this has been recently ascribed to the nature of the MP2 method [19]. Conversely, it was shown that when higher order coupling terms (*i.e.*, $\sum_{i<j<k}^{N}V_{i,j,k}^{triples}(Q_{i},Q_{j},Q_{k})$) are included in a DPT2-VSCF [44] treatment, MP2 indeed seems to be a viable *ab initio* method of choice [63]. VSCF data relies on the HOA and hence makes use of the rigid rotor approximation [70] which inherently is co-responsible for discrepancies between experiment and theory. Considering that still a large number of approximations are involved even in state-of-the-art VSCF calculations and their underlying *ab initio* energy evaluations, it must be concluded that computationally derived spectroscopic data are prone to a certain degree of fortuitous error compensation. However, both the data presented by Roy *et al.* [19] and our results indicate that triple-*ζ* based MP2 calculations in a VSCF treatment rather reliably deliver data with no more than 2% of unsigned error.

**Table 1:** Computed and experimental absorption data and the calculated values for *µ* in %. n.a. means “not applicable” and n.o. means “not observed”.

H_2_O (C_2*v*_)	Sym.	6	7	8	9	10	11	12	13	14	15	16	Expt. [59,71,72]
*ν*_*OH*_2_ ,*as*_	B_2_	2875	3997	3731	3772	3766	3766	3765	3765	3765	3765	3765	3756
*ν*_*OH*_2_ ,*s*_	A_1_	3041	3785	3657	3691	3684	3683	3683	3682	3682	3682	3682	3652
*δ*_*OH*_2__	A_1_	1216	1688	1570	1590	1586	1586	1586	1586	1586	1586	1586	1595
*μ*		21.3	5.3	0.8	0.6	0.6	0.6	0.5	0.5	0.5	0.5	0.5	n.a.
**CO_2_ (D_∞*h*_)**	**Sym.**	**6**	**7**	**8**	**9**	**10**	**11**	**12**	**13**	**14**	**15**	**16**	**Expt.** [59,73,74]
*ν*_*CO*_2_,*as*_	$\sum_{g}^{+}$	1791	2547	2359	2391	2386	2386	2385	2385	2385	2385	2385	2349
*ν*_*CO*_2_,*s*_	$\sum_{u}^{+}$	993	1408	1255	1319	1316	1315	1315	1315	1315	1315	1315	1285
*δ*_*CO*_2__	∏_*μ*_	487	699	645	654	653	653	653	653	653	653	653	667
*δ*_*CO*_2__	∏_*μ*_	487	699	645	654	653	653	653	653	653	653	653	667
*μ*		25.1	6.9	2.3	2.0	2.0	2.0	2.0	2.0	2.0	2.0	2.0	n.a.
**CH_2_O (C_2*v*_)**	**Sym.**	**6**	**7**	**8**	**9**	**10**	**11**	**12**	**13**	**14**	**15**	**16**	**Expt.** [59,75,76,77]
*ν*_*CH*_2_,*as*_	B_1_	2184	3000	2809	2837	2833	2833	2833	2832	2832	2832	2832	2843
*ν*_*CH*_2_,*s*_	A_1_	2250	2905	2801	2823	2818	2818	2818	2817	2817	2817	2817	2782
*ν_CO_*	A_1_	1312	1855	1719	1745	1741	1740	1740	1740	1740	1740	1740	1746
*δ*_*CH*_2__	A_1_	1157	1614	1502	1521	1518	1518	1518	1518	1518	1518	1518	1500
*ρ*_*CH*_2__	B_1_	945	1340	1240	1257	1254	1254	1254	1254	1254	1254	1254	1249
*ω*_*CH*_2__	B_2_	891	1250	1162	1176	1174	1173	1173	1173	1173	1173	1173	1167
*μ*		23.0	6.4	0.8	0.8	0.7	0.7	0.7	0.7	0.7	0.7	0.7	n.a.
**C_2_H_2_ (D_∞*h*_)**	**Sym.**	**6**	**7**	**8**	**9**	**10**	**11**	**12**	**13**	**14**	**15**	**16**	**Expt.** [59,60,78,79,80]
*ν*_*C*_2_*H*_2_,*s*_	$\sum_{g}^{+}$	2699	3490	3375	3390	3384	3380	3384	3384	3384	3384	3384	3374
*ν*_*C*_2_*H*_2_,*as*_	$\sum_{u}^{+}$	2523	3405	3199	3233	3228	3227	3227	3227	3227	3227	3227	3289
*ν_CC_*	$\sum_{g}^{+}$	1469	2046	1912	1937	1932	1932	1932	1932	1932	1932	1932	1974
*δ*_*C*_2_*H*_2_,*s*_	∏_*u*_	557	792	733	746	743	743	743	743	743	743	743	730
*δ*_*C*_2_*H*_2_,*s*_	∏_*u*_	557	792	733	746	743	743	743	743	743	743	743	730
*δ*_*C*_2_*H*_2_,*as*_	∏_*g*_	443	638	589	602	598	598	598	598	598	598	598	612
*δ*_*C*_2_*H*_2_,*as*_	∏_*g*_	443	638	589	602	598	598	598	598	598	598	598	612
*μ*		24.5	5.2	2.0	1.7	1.8	1.8	1.8	1.8	1.8	1.8	1.8	n.a.
**HCOOH (C_*s*_)**	**Sym.**	**6**	**7**	**8**	**9**	**10**	**11**	**12**	**13**	**14**	**15**	**16**	**Expt.** [59,81,82,83,84]
*ν_OH_*	A'	2841	3682	3530	3559	3550	3557	3552	3552	3552	3552	3552	3570
*ν_CH_*	A'	2246	3134	2959	2983	2978	2977	2977	2977	2977	2977	2977	2943
*ν_C=O_*	A'	1337	1910	1764	1790	1786	1786	1786	1785	1785	1785	1785	1770
*δ_CH_*	A'	1043	1480	1367	1384	1381	1381	1381	1381	1381	1381	1381	1387
*δ_OH_*	A'	982	1367	1265	1280	1277	1277	1277	1277	1277	1277	1277	1387
*ν_C-O_*	A'	848	1148	1077	1089	1086	1086	1086	1086	1086	1086	1086	1105
*δ_CH_*	A"	786	1114	1029	1042	1040	1040	1040	1040	1040	1040	1040	1033
*δ_OH,oop_*	A"	515	631	588	623	609	608	609	609	609	609	609	638
*δ_OCO_*	A'	469	659	611	619	617	617	617	617	617	617	617	625
*μ*		22.8	6.0	2.1	1.4	1.7	1.7	1.7	1.7	1.7	1.7	1.7	n.a.
**CH_4_ (T_*d*_)**	**Sym.**	**6**	**7**	**8**	**9**	**10**	**11**	**12**	**13**	**14**	**15**	**16**	**Expt.** [59,60,85,86]
*ν*_*CH*_3_,*as*_	F_2_	2333	3210	3000	3035	3029	3029	3028	3028	3028	3028	3028	3019
*ν*_*CH*_3_,*as*_	F_2_	2299	3177	2990	3020	3015	3015	3014	3014	3014	3014	3014	3019
*ν*_*CH*_3_,*as*_	F_2_	2330	3209	2999	3033	3028	3027	3027	3027	3027	3027	3027	3019
*ν*_*CH*_4_,*s*_	A_1_	2588	2968	2948	2943	2945	2944	2943	2943	2943	2943	2943	2917
*δ*_*CH*_4_,*as*_	E	1166	1641	1523	1542	1539	1539	1539	1539	1539	1539	1539	1534
*δ*_*CH*_4_,*as*_	E	1166	1641	1523	1542	1539	1539	1539	1539	1539	1539	1539	1534
*δ*_*CH*_3_,*s*_	F_2_	993	1390	1293	1309	1306	1306	1306	1306	1306	1306	1306	1306
*δ*_*CH*_3_,*s*_	F_2_	992	1390	1293	1309	1306	1306	1306	1306	1306	1306	1306	1306
*δ*_*CH*_3_,*s*_	F_2_	993	1390	1294	1309	1307	1307	1307	1306	1306	1306	1306	1306
*µ*		22.3	5.9	0.9	0.4	0.3	0.3	0.3	0.3	0.3	0.3	0.3	n.a.
**CH**_3_**Cl (C**_3*v*_**)**	**Sym.**	**6**	**7**	**8**	**9**	**10**	**11**	**12**	**13**	**14**	**15**	**16**	**Expt.** [59,87,88,89,90]
*ν*_*CH*_3_,*as*_	E	2338	3203	3002	3035	3030	3029	3029	3029	3029	3029	3029	3039
*ν*_*CH*_3_,*as*_	E	2306	3192	2974	3019	3016	3015	3015	3015	3015	3015	3014	3039
*ν*_*CH*_3_,*s*_	A1	2339	3074	2964	2989	2987	2986	2986	2985	2985	2985	2985	2937
*δ*_*CH*_3_,*as*_	E	1106	1555	1445	1463	1460	1460	1460	1460	1460	1460	1460	1452
*δ*_*CH*_3_,*as*_	E	1106	1556	1444	1462	1460	1459	1459	1459	1459	1459	1459	1452
*δ*_*CH*_3_,*s*_	A1	1036	1464	1357	1375	1372	1372	1371	1371	1371	1371	1371	1355
*ρ*_*CH*_3__	E	773	1099	1017	1031	1028	1028	1028	1028	1028	1028	1028	1017
*ρ*_*CH*_3__	E	776	1099	1020	1033	1031	1031	1031	1031	1031	1031	1031	1017
*ν_CCl_*	A1	585	802	749	760	758	758	758	758	758	758	758	732
*µ*		23.0	7.0	0.9	1.4	1.2	1.2	1.2	1.2	1.2	1.2	1.2	n.a.
**CH**_3_**OH (C**_*s*_**)**	**Sym.**	**6**	**7**	**8**	**9**	**10**	**11**	**12**	**13**	**14**	**15**	**16**	**Expt.** [59,60,91,92,93]
ν_OH_	A’	2812	3900	3646	3694	3687	3686	3686	3686	3686	3686	3686	3681
*ν*_*CH*_3_,*as*_	A’	2301	3198	2984	3035	3030	3029	3028	3028	3028	3028	3028	3000
*ν*_*CH*_3_,*as*_	A”	2230	3091	2881	2912	2908	2908	2907	2907	2907	2907	2907	2960
*ν*_*CH*_3_,*s*_	A’	2363	2951	2849	2957	2947	2942	2941	2941	2941	2941	2941	2844
*δ*_*CH*_3_,*as*_	A’	1130	1587	1475	1493	1490	1490	1490	1490	1490	1490	1490	1477
*δ*_*CH*_3_,*as*_	A”	1116	1574	1459	1478	1475	1475	1475	1475	1474	1474	1474	1477
*δ*_*CH*_3_,*s*_	A’	1104	1563	1447	1466	1463	1463	1463	1463	1463	1463	1463	1455
*δ*_*OH*_	A’	1020	1458	1342	1362	1359	1359	1358	1358	1358	1358	1358	1345
*ρ*_*CH*_3__	A”	843	1178	1069	1083	1081	1081	1080	1080	1080	1080	1080	1060
*ν*_*CO*_	A’	789	1060	1015	1028	1026	1026	1025	1025	1025	1025	1025	1033
*µ*		22.9	6.4	0.9	1.3	1.2	1.2	1.2	1.2	1.1	1.1	1.1	n.a.
**CH**_3_**CN (C**_3*v*_**)**	**Sym.**	**6**	**7**	**8**	**9**	**10**	**11**	**12**	**13**	**14**	**15**	**16**	**Expt.** [59,94,95,96,97,98,99,100]
*ν*_*CH*_3_,*as*_	E	2281	3153	2940	2972	2967	2967	2967	2966	2966	2966	2966	3009
*ν*_*CH*_3_,*as*_	E	2322	3172	2976	2994	2990	2989	2989	2988	2988	2988	2988	3009
*ν*_*CH*_3_,*s*_	A_1_	2322	3059	2951	2970	2968	2967	2967	2967	2967	2967	2967	2954
*ν_CN_*	A_1_	1656	2308	2152	2178	2174	2174	2173	2173	2173	2173	2173	2267
*δ*_*CH*_3_,*as*_	E	1099	1547	1438	1456	1453	1453	1453	1453	1453	1453	1453	1454
*δ*_*CH*_3_,*as*_	E	1099	1550	1437	1456	1453	1453	1453	1453	1453	1453	1453	1454
*δCH*3 ,*s*	A_1_	1047	1487	1378	1397	1394	1394	1394	1393	1393	1393	1393	1389
*ρ*_*CH*_3__	E	788	1121	1037	1052	1049	1049	1049	1049	1049	1049	1049	1041
*ρ*_*CH*_3__	E	789	1122	1038	1053	1050	1050	1050	1050	1050	1050	1050	1041
*ν_CC_*	A_1_	692	983	923	932	930	930	930	930	930	930	930	920
*δ_CCN_*	E	270	395	361	367	366	366	366	366	366	366	366	361
*δ_CCN_*	E	268	395	359	365	364	364	364	364	364	364	364	361
*µ*		24.4	6.4	1.1	1.1	1.0	1.0	1.0	1.0	1.0	1.0	1.0	n.a.
C_2_H_4_(D_2*h*_)	Sym.	6	7	8	9	10	11	12	13	14	15	16	Expt. [59,60,101,102]
*ν*_*CH*_2_,*as*_	B_2*u*_	2399	3358	3122	3161	3155	3154	3154	3154	3154	3154	3154	3106
*ν*_*CH*_2_,*as*_	B_1*g*_	2379	3328	3095	3133	3127	3126	3126	3126	3126	3126	3126	3103
*ν*_*CH*_2_,*s*_	A_*g*_	2297	3152	3047	3055	3052	3051	3051	3051	3051	3051	3051	3026
*ν*_*CH*_2_,*s*_	B_3*u*_	2318	3253	3023	3060	3055	3054	3054	3054	3054	3054	3054	2989
*ν_CC_*	A_*g*_	1273	1750	1628	1648	1644	1644	1644	1644	1644	1644	1644	1623
*δ*_*CH*_2__	B_3*u*_	1095	1554	1439	1458	1455	1455	1455	1455	1455	1455	1455	1444
*δ*_*CH*_2__	A_*g*_	1051	1432	1345	1361	1359	1358	1358	1358	1358	1358	1358	1342
*ρ*_*CH*_2__	B_1*g*_	922	1318	1217	1235	1232	1232	1232	1232	1232	1232	1232	1236
*τ*_*CH*_2__	A_*u*_	795	1129	1046	1060	1058	1058	1058	1058	1058	1058	1058	1023
*ω*_*CH*_2__	B_1*u*_	730	1036	960	973	971	971	971	971	971	971	971	949
*ω*_*CH*_2__	B_2*g*_	712	1013	939	951	949	949	949	949	949	949	949	943
*ρ*_*CH*_2__	B_2*u*_	619	893	824	836	834	834	834	834	834	834	834	826
*µ*		23.4	7.7	0.8	1.5	1.4	1.4	1.3	1.3	1.3	1.3	1.3	n.a.
**C**_2_**H**_4_**O (C**_2*v*_**)**	**Sym.**	**6**	**7**	**8**	**9**	**10**	**11**	**12**	**13**	**14**	**15**	**16**	**Expt.** [59,60,103,104]
*ν*_*CH*_2_,*as*_	B_2_	2372	3313	3083	3120	3114	3114	3113	3113	3113	3113	3113	3065
*ν*_*CH*_2_,*as*_	A_2_	2361	3296	3067	3104	3098	3098	3097	3097	3097	3097	3097	n.o.
*ν*_*CH*_2_,*s*_	A_1_	2378	3097	2939	3047	3064	3058	3057	3056	3056	3056	3056	3018
*ν*_*CH*_2_,*s*_	B_1_	2295	3214	2988	3024	3019	3018	3018	3018	3018	3018	3018	3006
*δ*_*CH*_2_,*s*_	A_1_	1153	1634	1500	1518	1515	1515	1515	1515	1515	1515	1515	1498
*δ*_*CH*_2_,*as*_	B_1_	1121	1590	1472	1492	1489	1489	1489	1489	1489	1489	1489	1472
*ν_CC_*	A_1_	1070	1328	1277	1288	1286	1285	1285	1285	1285	1285	1285	1270
*ρ*_*CH*_2__	A_2_	876	1253	1157	1174	1171	1171	1171	1171	1171	1171	1171	n.o.
*τ*_*CH*_2__	B_2_	871	1244	1149	1166	1163	1163	1163	1163	1163	1163	1163	1142
*ω*_*CH*_2__	B_1_	859	1229	1135	1152	1149	1149	1149	1149	1149	1149	1149	1151
*ω*_*CH*_2__	A_1_	882	1203	1128	1141	1139	1138	1138	1138	1138	1138	1138	1148
*τ*_*CH*_2__	A_2_	783	1117	1032	1047	1044	1044	1044	1044	1044	1044	1044	n.o.
*ν*_*OC*_2_,*s*_	A_1_	669	946	877	889	887	887	887	887	887	887	887	877
*ν*_*OC*_2_,*as*_	B_1_	624	891	822	833	832	831	831	831	831	831	831	872
*ρ*_*CH*_2__	B_2_	615	885	816	829	827	827	827	827	827	827	827	821
*µ*		23.3	6.5	1.2	1.4	1.4	1.3	1.3	1.3	1.3	1.3	1.3	n.a.
**CH**_3_**NH**_2_**(C**_*s*_**)**	**Sym.**	**6**	**7**	**8**	**9**	**10**	**11**	**12**	**13**	**14**	**15**	**16**	**Expt.** [59,105,106,107]
*ν*_*NH*_2_,*as*_	A”	2629	3517	3318	3348	3343	3343	3343	3343	3343	3343	3343	3411
*ν*_*NH*_2_,*s*_	A’	2539	3431	3362	3352	3347	3347	3346	3346	3346	3346	3346	3349
*ν*_*CH*_3_,*as*_	A”	2283	3110	2919	2947	2943	2942	2942	2942	2942	2942	2942	2985
*ν*_*CH*_3_,*as*_	A’	2283	3079	2907	2960	2957	2956	2956	2956	2955	2955	2955	2963
*ν*_*CH*_3_,*s*_	A’	2201	3010	2833	2889	2911	2906	2904	2904	2904	2904	2904	2816
*δ*_*NH*_2__	A’	1223	1733	1596	1610	1608	1608	1608	1608	1608	1608	1608	1642
*δ*_*CH*_3_,*as*_	A”	1132	1601	1483	1503	1500	1494	1500	1500	1500	1500	1500	1481
*δ*_*CH*_3_,*as*_	A’	1119	1581	1467	1487	1484	1483	1483	1483	1483	1483	1483	1463
*δ*_*CH*_3_,*s*_	A’	1083	1534	1421	1440	1437	1437	1436	1436	1436	1436	1436	1450
*ρ*_*CH*_3__	A”	998	1420	1314	1331	1328	1328	1328	1328	1328	1328	1328	n.o.
*ρ*_*CH*_3__	A’	891	1237	1152	1169	1167	1167	1167	1167	1167	1167	1167	1144
*ν_CN_*	A’	845	1109	1045	1059	1057	1057	1057	1057	1057	1057	1057	1050
*τ*_*NH*_2__	A”	725	1052	967	981	979	979	979	979	979	979	979	n.o.
*ω*_*NH*_2__	A’	637	909	840	852	851	850	850	850	850	850	850	816
*CN axis torsion*	A”	245	316	345	337	307	313	316	313	313	314	314	304
*µ*		22.8	5.9	2.4	2.3	1.6	1.7	1.8	1.7	1.7	1.7	1.7	n.a.
**C_2_H_6_(D_3*d*_)**	**Sym.**	**6**	**7**	**8**	**9**	**10**	**11**	**12**	**13**	**14**	**15**	**16**	**Expt.** [59,60,61,62,108,109]
*ν*_*CH*_3_,*as*_	E_*u*_	2309	3221	2999	3037	3031	3030	3030	3030	3030	3030	3030	2985
*ν*_*CH*_3_,*as*_	E_*g*_	2290	3185	2967	3011	3007	3006	3006	3006	3006	3006	3006	2969
*ν*_*CH*_3_,*s*_	A_1*g*_	2470	3006	2950	2981	3004	2996	2995	2995	2994	2994	2994	2954
*ν*_*CH*_3_,*s*_	A_2*u*_	2250	3172	2943	2982	2976	2975	2975	2975	2975	2975	2975	2896
*δ*_*CH*_3_,*as*_	E_*g*_	1123	1596	1474	1494	1491	1491	1491	1491	1491	1491	1491	1468
*δ*_*CH*_3_,*as*_	E_*u*_	1123	1593	1475	1496	1493	1493	1492	1492	1492	1492	1492	1469
*δ*_*CH*_3_,*s*_	A_1*g*_	1059	1501	1393	1411	1408	1408	1408	1408	1408	1408	1408	1388
*δ*_*CH*_3_,*s*_	A_2*u*_	1042	1482	1372	1392	1389	1389	1388	1388	1388	1388	1388	1379
*ρ*_*CH*_3__	E_*g*_	909	1294	1197	1215	1212	1212	1212	1212	1212	1212	1212	1190
*ν_CC_*	A_1*g*_	818	1051	1003	1010	1008	1008	1008	1008	1008	1008	1008	995
*ρ*_*CH*_3__	E_*u*_	620	899	828	842	839	839	839	839	839	839	839	822
*µ*		22.3	7.5	0.6	1.7	1.6	1.6	1.6	1.6	1.6	1.6	1.6	n.a.
**CH**_3_**OCH**_3_**(C**_2*v*_**)**	**Sym.**	**6**	**7**	**8**	**9**	**10**	**11**	**12**	**13**	**14**	**15**	**16**	**Expt.** [59,60,110,111]
*ν*_*CH*_3_,*as*_	A_1_	2339	3142	2945	3016	3013	3012	3012	3011	3011	3011	3011	2996
*ν*_*CH*_3_,*as*_	B_1_	2313	3145	2949	2976	2976	2976	2975	2975	2975	2975	2975	2996
*ν*_*CH*_3_,*as*_	A_2_	2242	3126	2906	2942	2936	2936	2936	2936	2936	2936	2935	2952
*ν*_*CH*_3_,*as*_	B_2_	2236	3117	2899	2934	2928	2928	2928	2928	2928	2928	2928	2925
*ν*_*CH*_3_,*s*_	A_1_	2206	2949	2825	2869	2852	2856	2855	2855	2855	2855	2854	2817
*ν*_*CH*_3_,*s*_	B_1_	2200	3080	2861	2897	2891	2891	2891	2891	2891	2891	2891	2817
*δ*_*CH*_3_,*as*_	A_1_	1142	1597	1488	1506	1503	1503	1503	1503	1503	1503	1503	1464
*δ*_*CH*_3_,*as*_	B_2_	1122	1587	1475	1494	1492	1491	1491	1491	1491	1491	1491	1464
*δ*_*CH*_3_,*as*_	B_1_	1118	1589	1475	1494	1491	1491	1491	1491	1491	1491	1491	1464
*δ*_*CH*_3_,*as*_	A_2_	1111	1579	1466	1485	1482	1482	1482	1482	1482	1482	1482	1464
*δ*_*CH*_3_,*s*_	A_1_	1114	1574	1460	1479	1476	1475	1475	1475	1475	1475	1475	1452
*δ*_*CH*_3_,s_	B_1_	1082	1541	1426	1445	1442	1442	1442	1442	1442	1442	1442	1452
*ρ*_*CH*_3__	A_1_	944	1349	1248	1265	1263	1262	1262	1262	1262	1262	1262	1244
*ρ*_*CH*_3__	B_1_	897	1275	1181	1197	1194	1194	1194	1194	1194	1194	1194	1227
*ρ*_*CH*_3__	B_2_	893	1273	1180	1194	1195	1190	1189	1188	1188	1188	1188	1179
*ρ*_*CH*_3__	A_2_	871	1245	1153	1168	1166	1166	1166	1166	1166	1166	1166	1150
*ν*_*C*_2_*O,as*_	B_1_	839	1207	1113	1129	1127	1127	1126	1126	1126	1126	1126	1102
*ν*_*C*_2_*O,s*_	A_1_	780	988	941	950	949	949	949	948	948	948	948	928
*δ*_*C*_2_*O*_	A_1_	333	452	423	429	428	428	428	428	428	428	428	418
*µ*		23.0	7.2	1.1	1.7	1.6	1.5	1.5	1.5	1.5	1.5	1.5	n.a.
**C**_2_**H**_5_**NO**_2_**(C**_*s*_**)**	**Sym.**	**6**	**7**	**8**	**9**	**10**	**11**	**12**	**13**	**14**	**15**	**16**	**Expt.** [112,113]
*ν_OH_*	A’	2773	3737	3524	3557	3552	3551	3551	3550	3550	3550	3550	3560
*ν*_*NH*_2_,*as*_	A”	2609	3556	3337	3370	3365	3364	3364	3364	3364	3364	3364	3410
*ν*_*NH*_2_,*s*_	A’	2629	3480	3346	3357	3352	3351	3351	3351	3351	3350	3350	n.o.
*ν*_*CH*_2_,*as*_	A”	2268	3101	2907	2937	2932	2932	2932	2931	2931	2931	2931	n.o.
*ν*_*CH*_2_,*s*_	A’	2265	3059	2982	2957	2953	2952	2952	2952	2952	2952	2952	2958
*ν_C=O_*	A’	1346	1921	1777	1802	1798	1798	1798	1798	1798	1798	1798	1779
*δ*_*NH*_2__	A’	1228	1750	1602	1620	1617	1617	1617	1617	1617	1617	1617	1630
*δ*_*CH*_2__	A’	1085	1532	1421	1440	1437	1437	1437	1437	1437	1437	1437	1429
*ν*_*C−O*_	A’	1043	1482	1372	1391	1388	1388	1388	1387	1387	1387	1387	1373
*τ*_*CH*_2_,*NH*_2__	A”	1030	1466	1355	1374	1371	1371	1371	1371	1370	1370	1370	n.o.
*ω*_*CH*_2__	A’	968	1382	1275	1293	1290	1290	1290	1290	1290	1290	1290	n.o.
*δ_CCN,oop_*	A”	881	1262	1164	1181	1178	1178	1178	1178	1178	1178	1178	n.o.
*ν_CN_*	A’	905	1245	1156	1172	1169	1169	1169	1169	1169	1169	1169	1136
*δ*_*CO*_2__	A’	832	1186	1097	1113	1110	1110	1110	1110	1110	1110	1110	1101
*δ*_*CNH*_2__	A’	716	1002	922	938	935	935	934	934	934	934	934	907
*δ*_*C=O,oop*_	A”	685	990	910	924	922	922	922	922	922	922	922	883
*ν*_*C −C*_	A’	658	863	815	823	822	822	822	822	822	822	822	801
*δ*_*C=O,ip*_	A’	486	678	629	637	636	636	636	636	636	636	636	619
*C-O axis torsion*	A”	369	529	475	485	483	483	483	483	483	483	483	500
*O-C*_2_*N axis shear*	A’	365	495	461	467	466	466	466	466	466	466	466	463
*O=C*_2_*N axis shear*	A’	194	286	262	266	266	266	266	266	266	266	266	n.o.
*µ*		22.8	7.5	1.5	1.8	1.7	1.7	1.7	1.7	1.7	1.7	1.7	n.a.

### 3.2. Performance at Reduced Grid Densities

As a VSCF evaluation with 16 grid points per mode is computationally highly demanding, a reduction of the grid density is desirable. From the data in Table 1 it becomes evident that for most cases, a reduction to 10 grid points yields identical results as the corresponding calculation with 16 grid points, indicating that the computational demand in a VSCF treatment of $\sum_{i=1}^{N}V_{i}^{diag}(Q_{i})$ and $\sum_{i<j}^{N}V_{i,j}^{pairs}(Q_{i},Q_{j})$ contributions can be reduced safely by more than 50%. It has to be kept in mind, however, that for every set of reduced densities, the grid is interpolated to the original resolution and the displacement boundaries are not affected by the reduction. Hence, as long as the points on the potential energy surface are chosen properly, the accuracy of the VSCF results is not affected. Noteworthy, the default routine in GAMESS chooses the displacement boundaries in dependence to the underlying normal mode’s wavenumber but the user is nonetheless able to alter these settings if desired.

**Table 2:** Obtained values for *µ* in %, their arithmetic means and the corresponding *t*-values according to Equation (12). n.a. means “not applicable”.

Number of Grid Points											
Molecule	6	7	8	9	10	11	12	13	14	15	16
**H_2_O**	21.3	5.3	0.8	0.6	0.6	0.6	0.5	0.5	0.5	0.5	0.5
**CO_2_**	25.1	6.9	2.3	2.0	2.0	2.0	2.0	2.0	2.0	2.0	2.0
**CH_2_O**	23.0	6.4	0.8	0.8	0.7	0.7	0.7	0.7	0.7	0.7	0.7
**C_2_H_2_**	24.5	5.2	2.0	1.7	1.8	1.8	1.8	1.8	1.8	1.8	1.8
**HCOOH**	22.8	6.0	2.1	1.4	1.7	1.7	1.7	1.7	1.7	1.7	1.7
**CH**_4_	22.3	5.9	0.9	0.4	0.3	0.3	0.3	0.3	0.3	0.3	0.3
**CH**_3_**Cl**	23.0	7.0	0.9	1.4	1.2	1.2	1.2	1.2	1.2	1.2	1.2
**CH**_3_**OH**	22.9	6.4	0.9	1.3	1.2	1.2	1.2	1.2	1.1	1.1	1.1
**CH**_3_**CN**	24.4	6.4	1.1	1.1	1.0	1.0	1.0	1.0	1.0	1.0	1.0
**C**_2_**H**_4_	23.4	7.7	0.8	1.5	1.4	1.4	1.3	1.3	1.3	1.3	1.3
**C**_2_**H**_4_**O**	23.3	6.5	1.2	1.4	1.4	1.3	1.3	1.3	1.3	1.3	1.3
**CH**_3_**NH**_2_	22.8	5.9	2.4	2.3	1.6	1.7	1.8	1.8	1.7	1.7	1.7
**C**_2_**H**_6_	22.3	7.5	0.6	1.7	1.6	1.6	1.6	1.6	1.6	1.6	1.6
**CH**_3_**OCH**_3_	23.0	7.2	1.1	1.7	1.6	1.5	1.5	1.5	1.5	1.5	1.5
**C**_2_**H**_5_**NO**_2_	22.8	7.5	1.5	1.8	1.7	1.7	1.7	1.7	1.7	1.7	1.7
**Mean** *µ*	23.1	6.5	1.3	1.4	1.3	1.3	1.3	1.3	1.3	1.3	1.3
***t*-Value**	102.96	25.59	0.09	2.43	0.81	0.78	1.71	1.34	0.66	2.39	n.a.

Reviewing the values for *µ* in Table 2 permits the conclusion that major errors are starting to appear at grid densities <8 points. The GAMESS manual states that the VSCF code is “thought to give accuracy to 50 cm^−1^ for the larger fundamentals” when MP2 with a triple-*ζ* basis set is employed and hence, the boundaries of accuracy are wider than the error inflicted by a reduction of the grid density to 8 points. In order to provide statistical evidence as to what extent a reduction of the grid density is viable, paired t-tests [114] of the obtained values for *µ* have been conducted with the following equation:

(12) $$t=\frac{\left|\sum_{i=1}^{\chi }\frac{\mu_{ref}-\mu_{calc}}{\chi }\right|}{s_{d}}\times \sqrt{\chi }$$

In Equation (12), *µ*_*ref*_ is the reference value at a grid density of 16 points. *µ*_*calc*_ is the mean absolute percentage deviation at the examined grid density. *χ* is the number of investigated molecules and the *t*-value is calculated with *s*_*d*_ as the corrected sample standard deviation of the respective grid density. For a two-tailed problem, critical values for *t* can be specified according to Table 3.

**Table 3:** Critical boundaries according to Student’s t-distribution for a two-tailed problem.

*t*-Value	Difference
t ≤ 2.145	Insignificant
2.145 < t ≤ 2.977	Probable
2.977 < t ≤ 4.140	Significant
4.140 < t	Highly significant

Except for the data obtained at grid densities of 6 and 7 points, no significant deviations from the reference data are observed, indicating that even a reduction to 8 grid points is a very viable option. The computational effort involved in a pair-approximated VSCF evaluation (Equation (7)) may thus be reduced by a factor of nearly 4. It has to be stressed, however, that the obtained *t*-value for *r* = 8 (*i.e.*, 0.09) may not be confused as to indicate the objectively best agreement with the reference values since the statistical analysis is based on unsigned error values. Moreover, the anharmonic correction seems to be slightly over-estimated at *r* = 8, yielding more red-shifted absorptions, as can be learned from Table 1. This over-correction is responsible for a slightly improved agreement with experiment for most of the molecules, especially since it is known that MP2 tendentially yields blue-shifted IR absorptions [19]. Overall, it may be concluded that VSCF treatments involving at least 8 grid points reproduce the experimental values within the same boundaries of accuracy as the reference values with a mean absolute percentage deviation from experiment of ~1.3% (Table 2).

In their recent paper, Roy *et al.*, reported that soft and semi-rigid molecules have to be treated at higher grid densities than rigid molecules and they mention ethylene oxide as a particular example where the VSCF equations converge at no less than 12 grid points. Our results contradict this statement since our VSCF calculations of ethylene oxide did indeed converge for grid densities ≥6 points and a density of 8 points yielded the same quality of results as observed for the other molecules. Given the fact that the identical code, the same *ab initio* method and the same basis set were employed, this outcome is rather puzzling. This disagreement may be ascribed to the use of symmetry during our VSCF calculations and the underlying reference geometries having been minimized to the most stringent criterion available. Overall, none of our VSCF evaluations exhibited convergence problems apart from the few excluded torsional degrees of freedom that have been mentioned earlier.

### 3.3. Application to Deprotonated Phosphoserine

**Figure 1:**
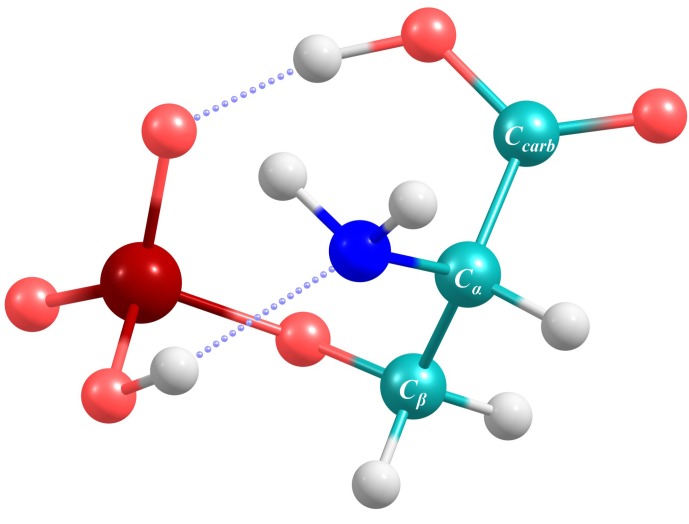
The lowest energy conformer of [pSer-H]^−^ exhibits a hydrogen bond between the carboxylic OH and the phosphate O and between the phosphate OH and the amino N.

**Figure 2:**
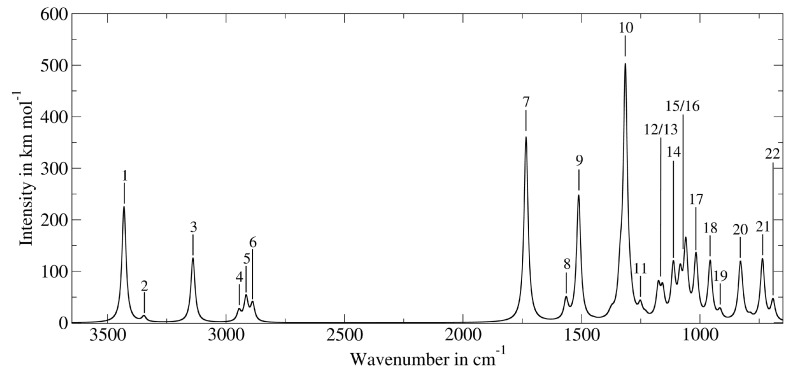
The computed IR spectrum of [pSer-H]^−^. For band assignments, refer to Table 4.

Phosphorylation of proteins is considered a major signal transduction mechanism, mainly occurring at the OH terminus of the amino acids serine, threonine and tyrosine. As a major constituent of biofluids, phosphoserine was subject of a gas-chromatographic investigation of human urine samples [115]. In autopsied Alzheimer’s disease brain tissue, l-phosphoserine was found in elevated concentrations [116] and only very recently, plasma phosphoserine levels were found to be upregulated in sepsis patients [117]. Deprotonated phosphoserine, abbreviated as [pSer-H]^−^, was investigated via infrared multiple photon dissociation (IRMPD) spectroscopy and hence, experimental data of a few major gas phase IR absorptions of the molecule is available [118]. The authors identified the lowest energy conformer of [pSer-H]^−^ as the one exhibiting hydrogen bonds between the carboxylic OH and the phosphate O as well as between the phosphate OH and the amino N. This conformer was chosen as a benchmark for the 8 point VSCF analysis due to the fact that it represents rigid and weak structural motifs likewise. Figure 1 shows the minimum geometry obtained at the MP2/cc-pVTZ level of theory which served as a reference state for the VSCF evaluation. Table 4 lists the PT2-VSCF computed absorptions of [pSer-H]^−^, the respective intensities and the available experimental data [118]. Due to the cage-like structure, many normal modes couple among each other and hence, especially lower vibrations cannot be ascribed to distinct normal modes. In such cases, the main vibrational contributions are given in Table 4. For illustrative purposes, the calculated IR spectrum of [pSer-H]^−^ is shown in Figure 2. The intensities have been computed using harmonic dipole derivatives at the MP2/cc-pVTZ level of theory and band broadening was introduced via a Lorentzian function using a band width at half height of 20 cm^−1^.

**Table 4:** The main absorptions of [pSer-H]^−^ and the experimentally obtained [118] values.

Band Number	PT2-VSCF (cm^−1^)	Intensity (km mol^−1^)	IRMPD (cm^−1^) [118]	Main Contributions
1	3430	225	n.o.	*ν*_*OH*_
2	3346	11	n.o.	*ν*_*NH*_2_*,as*_
3	3139	125	n.o.	*ν*_*NH*_2_*,s*_
4	2945	21	n.o.	*ν*_*C*_*α*_*H*_
5	2915	47	n.o.	*ν*_*C*_*β*_*H*_2_*,s*_
6	2888	35	n.o.	*ν*_*C*_*β*_*H*_2_*,as*_
7	1734	359	1728	*ν*_*C*_*carb*__ =*O*
8	1565	39	1610	*δ*_*NH*_2__
9	1512	244	1461	*δ*_*C*_*carb*_*OH,ip*_
	1449	1	1419	*δ*_*C*_*β*_*H*_2__
	1380	4	n.o.	*τ*_*NH*_2__ and *δ*_*NC*_*α*_*H*_
	1371	9	n.o.	*ω*_*C*_*β*_*H*_2__
	1337	68	n.o.	*ν*_*C*_*carb*_*−O*_ and *τ*_*C*_*β*_*H*_2__
10	1315	484	1291	*ν*_*P*_ =*O*
	1292	21	n.o.	*ω*_*C*_*β*_*H*_2__ and *δ*_*C*_*carb*_*C*_*α*_*H*_ and *ν*_*C*_*carb*_*−O*_
11	1251	24	n.o.	*ν*_*C*_*α*_ − *C*_*β*__ and *δ*_*C*_*β*_*C*_*α*_*H*_ and *ν*_*C*_*α*_*−N*_ and *τ*_*NH*_2__
	1230	6	n.o.	*ν*_*C*_*carb*_*C*_*α*__ and *δ*_*C*_*carb*_*C*_*α*_*H*_ and *τ*_*NH*_2__
12	1176	60	n.o.	*δ*_*C*_*carb*_*OH,oop*_
13	1157	51	n.o.	*ν*_*C*_*β*_*O*_
14	1113	99	1108	*δ*_*POH*_
15	1084	74	n.o.	*ν*_*NC*_*α*_*C*_*carb*_*,as*_ and *ν*_*C*_*β*_*O*_
16	1060	141	1052	*ω*_*NH*_2__
17	1017	121	1028	*ν*_*P − O*_*H −bonded*__
18	957	113	n.o.	*ν*_*NC*_*α*_*C*_*β*_*,as*_
19	915	17	n.o.	*ν*_*NC*_*α*_*C*_*β*_*,s*_ and *ν*_*NC*_*α*_*C*_*carb*_*,as*_ and *ν*_*C*_*carb*_*C*_*α*_*C*_*β*_*,as*_
20	830	98	836	*ν*_*P −OH*_
	823	24	812	*δ*_*C*_*carb*_*C*_*α*_*C*_*β*__ and *δ*_*C*_*carb*_*O*_2_*,oop*_
	787	7	n.o.	*δ*_*C*_*carb*_*O*_2__ and *δ*_*C*_*carb*_*C*_*α*_*C*_*β*__
21	736	120	738	*ν*_*P −OC*_*β*__ and *δ*_*C*_*carb*_*O*_2__
22	692	39	n.o.	*δ*_*C*_*α*_*NC*_*β*_*C*_*carb*_*,umbrella*_ and *ν*_*P − OC*_*beta*__
	513	41	n.o.	*δ*_*POH,oop*_

Considering the 11 available experimental values, *µ* was computed for the corresponding VSCF data as 1.38%. Since we did calculate the IR absorptions of [pSer-H]^−^ exclusively using 8 grid points per mode, no definitive conclusion can be drawn whether higher grid densities would enable an improved accuracy for this larger molecule. A re-evaluation of the potential energy surface at grid densities up to 16 points would be unmanageably expensive but nonetheless, the data presented fits well into the error boundaries as observed for the 15 molecules discussed earlier.

The band assignment in Table 4 clearly shows that empirical considerations are only applicable to the large fundamentals. For a proper description of low lying vibrations that are determined by a number of torsions, computational approaches are an important option aiding the spectroscopist. In their experimental work, Scuderi *et al.*, have employed computational techniques during their band assignment of [pSer-H]^−^ as well, but they resorted to scaled HOA absorption data at the B3LYP/6-311+G(d,p) level of theory.

## 4. Conclusions

VSCF theory has grown an important field in computational spectroscopy and this is owed to the increasingly available computational resources and to efficient and easily applicable algorithms. With this systematic study it has been demonstrated that a largely reduced grid density in a VSCF evaluation does not only spare considerable resources but also does not significantly affect the resulting absorption data. It was found that the convergence of VSCF equations is not impaired by such a reduced-effort technique even when highly problematic densities of <8 grid points are employed. While further investigations are required with regard to the role of symmetry in a VSCF treatment, it was found that reduced grid densities may be safely applied to a wide set of molecular entities. Application to [pSer-H]^−^ showed that a 8 point VSCF calculation yields accurate data for a molecule exhibiting weak interactions. The routine presented herein should, in conjunction with other recent developments [35], prove valuable for larger molecules relevant to life sciences where a conventional VSCF treatment with 16 grid points per mode is not feasible.
